# Ontogeny of different subsets of yellow fever virus-specific circulatory CXCR5^+^ CD4^+^ T cells after yellow fever vaccination

**DOI:** 10.1038/s41598-020-72610-6

**Published:** 2020-09-24

**Authors:** Quinn DeGottardi, Theresa J. Gates, Junbao Yang, Eddie A. James, Uma Malhotra, I-Ting Chow, Yannick Simoni, Michael Fehlings, Evan W. Newell, Hannah A. DeBerg, William W. Kwok

**Affiliations:** 1 Benaroya Research Institute At Virginia Mason Medical Center, 1201, 9th Ave, Seattle, WA 98101 USA; 2 Virginia Mason Hospital, Seattle, WA USA; 3 Vaccine and Infectious Disease Division, Fred Hutchinson Cancer Research Center, Seattle, WA USA; 4 Singapore Immunology Network, Agency for Science Research and Technology, Singapore, Singapore; 5 Department of Medicine, University of Washington, Seattle, WA USA; 6 Present Address: Adaptive Biotechnologies, Seattle, WA USA; 7 Present Address: Cs-Bay Therapeutics, Newark, CA USA

**Keywords:** Adaptive immunity, Vaccines, Immunology

## Abstract

Monitoring the frequency of circulatory CXCR5^+^ (cCXCR5^+^) CD4^+^ T cells in periphery blood provides a potential biomarker to draw inferences about T follicular helper (T_FH_) activity within germinal center. However, cCXCR5^+^ T cells are highly heterogeneous in their expression of ICOS, PD1 and CD38 and the relationship between different cCXCR5 subsets as delineated by these markers remains unclear. We applied class II tetramer reagents and mass cytometry to investigate the ontogeny of different subsets of cCXCR5^+^ T cell following yellow fever immunization. Through unsupervised analyses of mass cytometry data, we show yellow fever virus-specific cCXCR5 T cells elicited by vaccination were initially CD38^+^ICOS^+^PD1^+^, but then transitioned to become CD38^+^ICOS^−^PD1^+^ and CD38^−^ICOS^−^PD1^+^ before coming to rest as a CD38^−^ICOS^−^PD1^−^ subset. These results imply that most antigen-specific cCXCR5^+^ T cells, including the CD38^−^ICOS^−^PD1^−^ CXCR5^+^ T cells are derived from the CXCR5^+^CD38^+^ICOS^+^PD1^+^ subset, the subset that most resembles preT_FH_/T_FH_ in the germinal center.

## Introduction

CD4 T helper cells are highly heterogeneous and can be classified into different subsets based on their functions and cytokine production profiles. The major CD4^+^ T cell subsets include the Type 1 T helper (T_H1_), Type 2 T helper (T_H2_), Type 17 T helper (T_H17_), T follicular helper (T_FH_) and regulatory T cell (Treg) subsets^1^. Effective monitoring of T_FH_ cell responses after vaccination would be of great interest, as the activity of T_FH_ cells in germinal centers is essential for B cell affinity maturation and subsequent generation of the high affinity antibodies that are crucial for vaccine efficacy^2–4^. However, because T_FH_ cells are sequestered within secondary lymphoid tissues, monitoring these cells directly is largely impractical in human studies.

Pre-T_FH_ and T_FH_ cells which are found in the T cell-B cell interface and the germinal centers of secondary lymphoid tissues respectively are characterized by the elevated cell surface expression of CXCR5, ICOS, PD1 and intracellular expression of the lineage defining transcription factor BCL-6^2–4^. Approximately 20% of memory CD4^+^ T cells in peripheral human blood also express CXCR5^5,6^. Although these cells have been shown to induce B cells to differentiate into immunoglobulin producing plasmablasts in vitro^7,8^, some uncertainty remains as whether these circulating CXCR5^+^ (cCXCR5^+^) T cells are bona fide memory T_FH_ cells because they do not express Bcl-6 and the surface expression level of CXCR5 is much lower in comparison to T_FH_ cells in lymphoid tissues^5,8,9^. Surface expression levels of ICOS and/or PD1 are also lower or even absent on a significant proportion of cCXCR5^+^ T cells in peripheral blood.

Previous studies have examined whether cCXCR5^+^ T cells represent memory GC-T_FH_ cells that have exited GC of lymphoid tissues and migrated back to the periphery. Data from murine adoptive transfer experiments provided evidence that cCXCR5^+^ T cells are biased toward becoming T_FH_^10^. However, in a mouse model in which GC formation was impaired (mediated by a defective Sh2d1a gene), the frequencies of cCXCR5^+^ T cells were within the normal range^9^. Therefore, existing data suggests that cCXCR5^+^ T cells could be derived either from pre-GC-T_FH_ but does not rule out that cCXCR5^+^ T cells could be derived from GC-T_FH_. Human studies have shown that the frequencies of cCXCR5^+^ T cells are correlated with the robustness of the antibody responses^11,12^ and demonstrate sharing of TCR clonotypes between cCXCR5^+^ T cells and GC-T_FH_^13^. More recent data also show TCR clonotype sharing between cCXCR5^+^ T cells and thoracic duct CXCR5^+^ T cells and similarity between transcriptome and epigenetic profiles between GC-T_FH_ and thoracic duct CXCR5^+^, suggesting that GC-T_FH_ transit through the thoracic duct as CXCR5-bright PD1-bright T cells before entering the peripheral blood as cCXCR5^+^ T cells^14^.

Based on these studies, it can be surmised that examining cCXCR5^+^ CD4^+^ T cells would be a reasonable surrogate to draw inferences about the robustness of T_FH_ activity in GC. However, there is a remaining knowledge gap concerning the highly heterogeneous surface marker expression exhibited by cCXCR5^+^ T cells. In particular, given that cCXCR5^+^ cells exhibit various combinations of ICOS, PD1, CD38 and CCR7 expression, it is unclear which subsets of cCXCR5^+^ T cells would be the best indicator of T_FH_ activity in GC.

Some prior studies monitored the increase in all cCXCR5^+^ populations after vaccination^15^, whereas others examined the increase of PD1^+^^11^, PD1^hi^CCR7^lo^^9^, ICOS^+^CD38^+^^16^, PD1^+^ICOS^+^^13^, or ICOS^+^CXCR3^+^CD38^+^^12^ cCXCR5^+^ T cells after vaccination. All these subsets demonstrated apparent utility as biomarkers to reflect GC-T_FH_ responses, but the relationship between these apparently diverse cCXCR5^+^ T cell subset remains incompletely resolved. Other studies examined overlaps in TCR clonotype between different subsets of cCXCR5^+^ T cells, showing that the PD1^+^ICOS^+^CD38^+^ subset shared clonotypes with the PD1^+^ICOS^−^CD38^−^ subset^16^ and that the PD1^+^ICOS^+^ subset shared clonotypes with the PD1^+^ICOS^−^ subset^13^. These data were used to describe the lineage relationships between these different cCXCR5 subsets. All these published studies have relied on PD1, ICOS and CD38 to draw inferences about antigen specific cCXCR5^+^ T cells. As PD1, ICOS and CD38 are also T cell activation markers, the use of these markers to define linage relationship between these different subsets of antigen specific cCXCR5 T cells can confound the interpretation of the results.

Therefore, we set out to perform a comprehensive MHC class II tetramer based analysis of antigen specific cCXCR5^+^ T cells with the objective of refining our understanding of the relationship between different subsets by examining the ontogeny and kinetics of emergence following vaccination. Specifically, we applied metal labeled MHC class II tetramers and mass cytometry to monitor yellow fever virus (YFV)-specific CD4^+^ T cells in peripheral blood of human subjects after primary vaccination with YF-Vax (yellow fever vaccine), a live attenuated strain vaccine known to provide life-long protection^17^. Postulating that YF-Vax vaccination elicits a high frequency of YFV-specific cCXCR5^+^ T cells, we sought to test the hypothesis that the longitudinal development and ontogeny of YFV-specific cCXCR5^+^ T cells that emerges post vaccination would reveal meaningful relationships between different cCXCR5^+^ T cell subsets. To provide a meaningful comparator to YFV-specific CD4 T cell responses, Influenza, tetanus toxoid and EBV specific CD4^+^ T cells were also monitored over the course of the study.

## Results

### Mass cytometry analysis of antigen specific CD4^+^ T cell responses after vaccination

**Figure 1:**
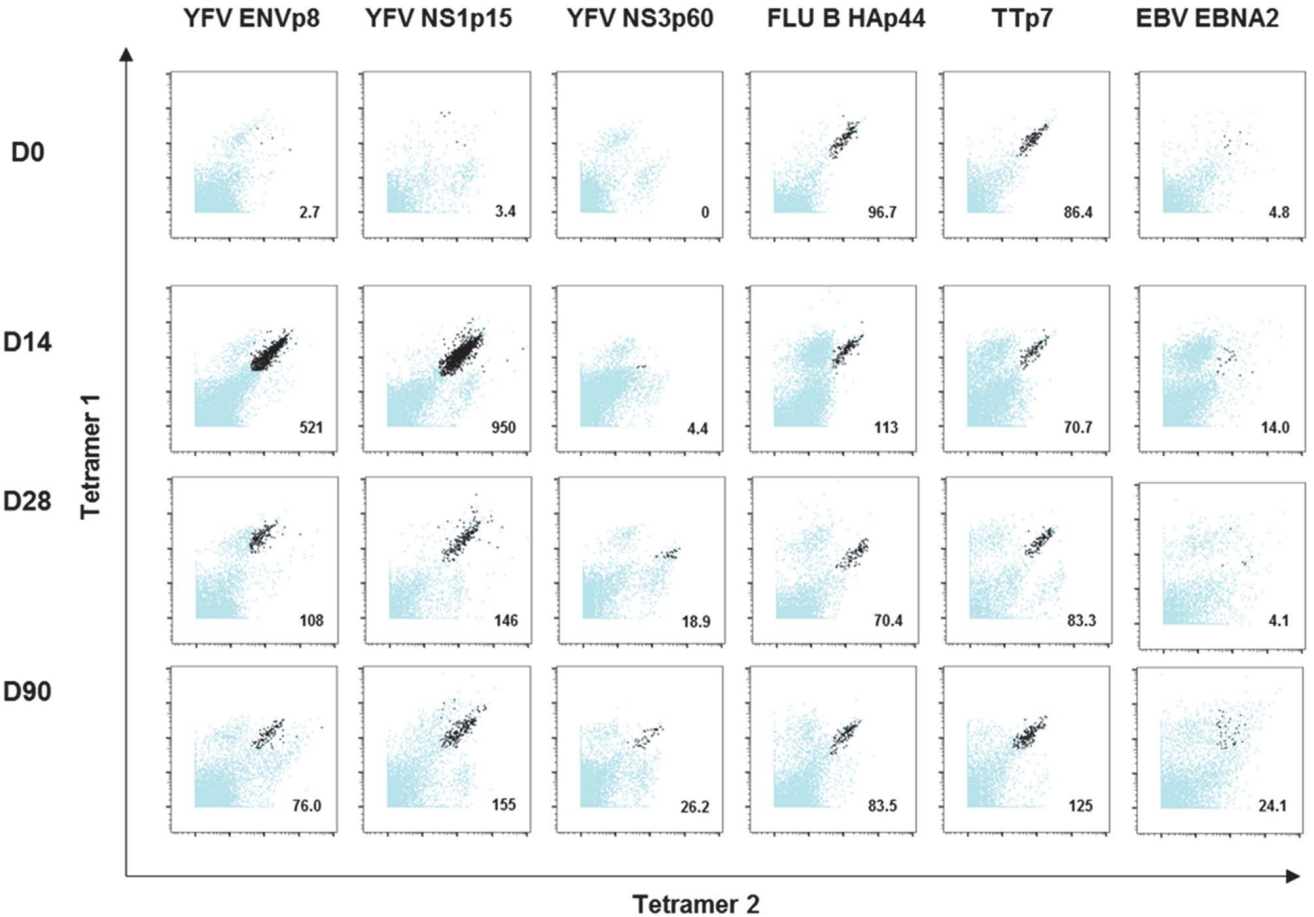
Combinatorial ex vivo class II tetramers staining of YFV-specific, TT-specific, FLU B-specific, EBV-specific CD4^+^ T cells at 4 different time points. Combinatorial tetramer staining was carried out in PBMCs from the same subject at day 0 (pre-vaccination) and day 14, 28 and 90 post YF-Vax vaccination. Tetramer for each epitope specificity was conjugated to two different metal tags as show in Table S1. Staining for specific T cells of different epitope specificities at each time point was carried out in a single tube. Dark dots represent epitope specific cells and blue dots represent other CD4^+^ T cells. Frequencies of epitope specific CD4^+^ T cell per million CD4^+^ T cells are as indicated.

**Figure 2:**
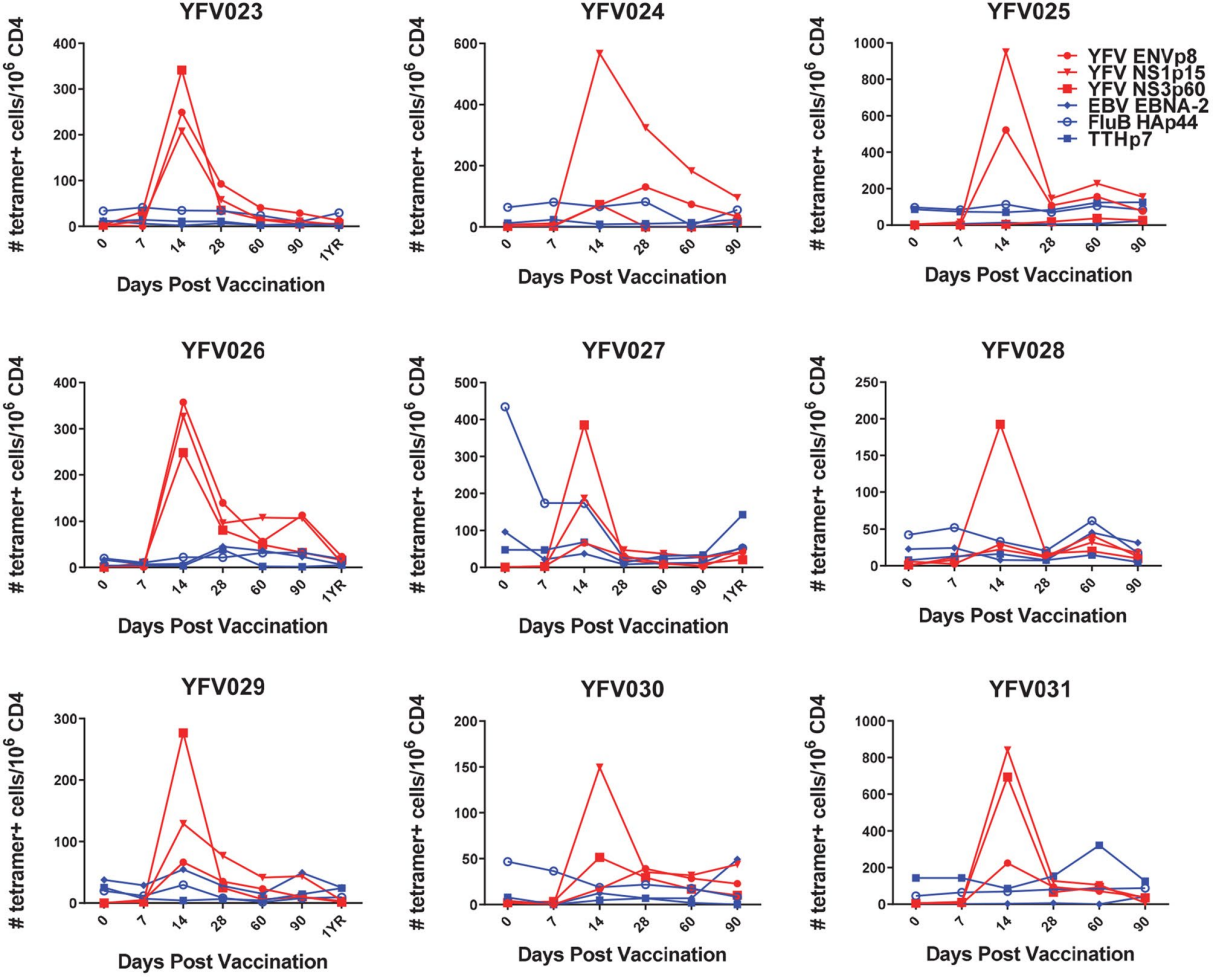
Kinetics of YFV-specific, TT-specific, FLU B-specific, EBV-specific CD4^+^ T cell responses post YF-Vax vaccination. PBMC from nine DRB1*0301 subjects were stained with 3 different YFV-specific tetramers, TT-specific, FLU B-specific and EBV-specific tetramers and a panel of 28 different antibodies as listed in Tables S1 and S2. Frequencies of CD45RO^+^ YFV ENV-specific, YFV NS-1-specific, YFV NS3-specific, TT, FLU B HA-specific and EBV EBNA2-specific CD4^+^ T cells of nine different subjects at different time points post YFV-Vax vaccination are shown.

Mass cytometry was used to examine antigen-specific CD4^+^ T cell responses after primary vaccination with YF-Vax in nine HLA-DRB1*03:01 healthy subjects. For four subjects, peripheral blood was obtained immediately before immunization and at 7, 14, 28, 60, 90 and 360 days after vaccination. For the remaining five subjects, samples were obtained only for the first six time points, but the long term 360 day sample was not obtained. A broad panel of metal labeled HLA-DRB1*03:01 tetramers containing YFV envelope (ENV), YFV non-structural protein 1 (NS1), YFV non-structural protein 3 (NS3), influenza B hemagglutinin (FLU B HA), tetanus toxoid (TT) and EBV EBNA2 epitopes was combined with a carefully selected antibody panel to monitor the frequency and surface phenotype of antigen specific CD4^+^ T cells in PBMCs at different time points. The specific tetramers and antibodies used are shown in Tables S1 and S2. Each of the YFV, TT and FLU B HA epitopes had been previously identified by Tetramer-guided epitope mapping^18,19^ and could be located in the IEDB database. The EBNA2 epitope had also been discovered and reported earlier^20^. Our gating strategy for identifying tetramer positive CD4^+^ T cells is shown in Fig. S1. Representative tetramer staining (for all epitopes at day 0, day 14, day 28 and day 90) from a single subject are shown in Fig. 1. The overall frequency of YFV specific T cells (YFV ENV, NS1, and NS3) peaked at day 14 after primary vaccination (Fig. 2). In contrast, the frequencies of TT-specific, FLU B HA-specific and EBV EBNA2-specific CD4^+^ T cells remained fairly stable, reflecting the fact that subjects were not immunized or exposed to these antigens over the course of our study (Fig. 2). The single exception was subject YFV027, who had a higher frequency of FLU B HA-specific CD4^+^ T cells at day 0, 7 and 14. The memory CD45RO^+^ FLU-B HA-specific CD4^+^ T cells of this subject at day 0 and day 14 also expressed high level of CD38, suggesting that there were recently activated T cells (Fig. S2). This was caused by influenza vaccination of this subject a few weeks prior to his/her enrollment in our study, as our prior work shows that recently activated influenza specific T cells express CD38 and their frequencies can remain elevated for at least 60 days following exposure^21^.

### UMAP analysis of total CD4^+^T cells, EBV, FLU, TT and YFV-specific CD4^+^ T cells

**Figure 3:**
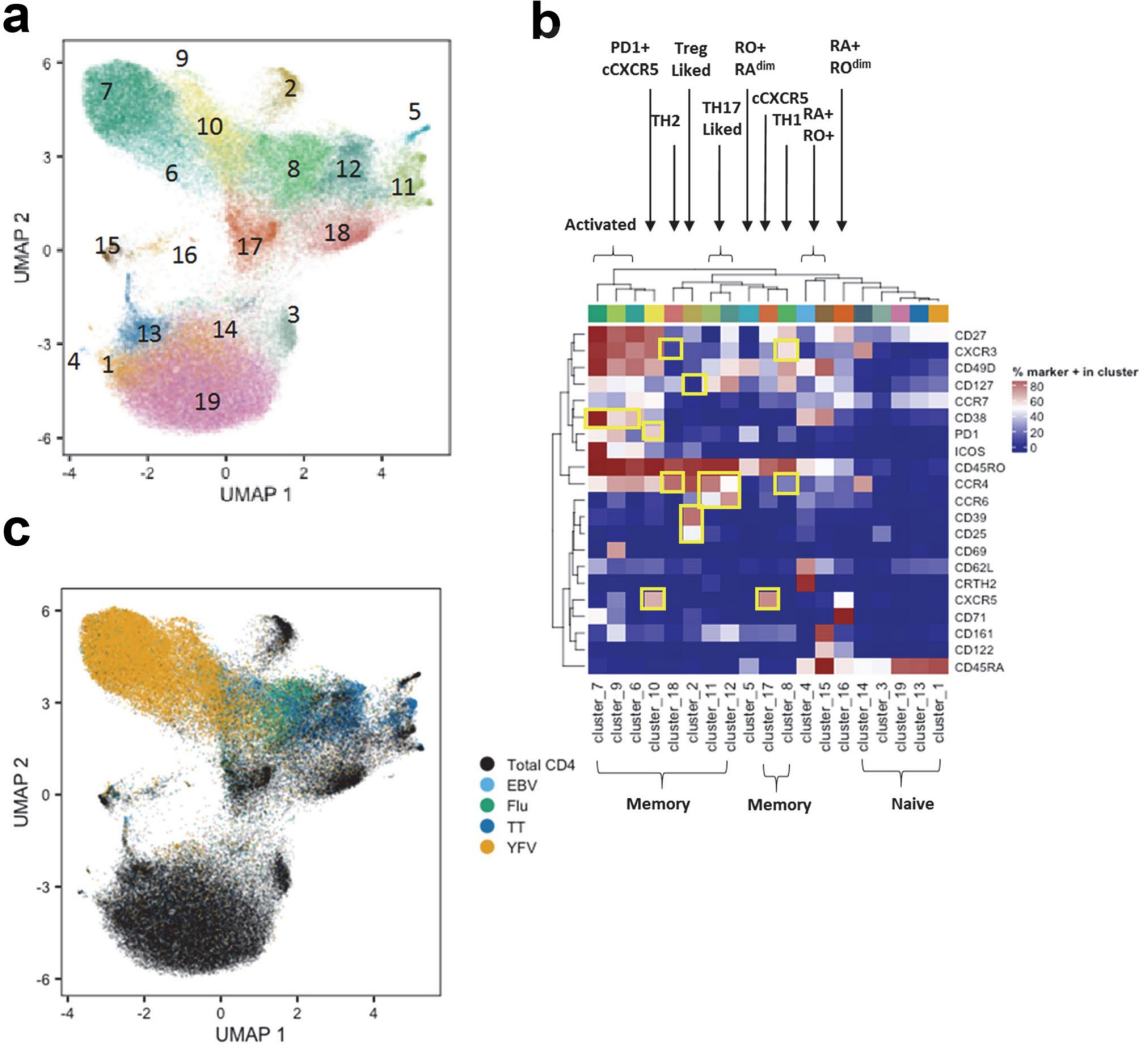
Cellular clustering of total CD4^+^ T cells and epitope specific CD4^+^ T cells pre and post YF-Vax vaccination. (**a**) UMAP was applied for visualization of the high dimensional CyTOF data set which examines the surface expression of 21 different markers in CD4^+^ T cells. Marker intensities were arcsinh transformed before further data analysis. To adjust for batch differences between samples, a z-score normalization was applied to each marker relative to the total CD4 T cell expression for that marker on the same subject and visit prior to dimensionality reduction using UMAP. Clustering was performed with a total of 82,811 CD4^+^ T cells from all 9 subjects at all time points assayed (a total of 58 samples), including 17,851 YFV-specific, 1280 EBV-specific, 2913 FLU-specific, 2767 TT-specific CD4^+^ T cells and from 58,000 non-YFV, non-EBV, non-FLU, and non-TT CD4^+^ T cells (1000 cells from each subject at each time point). PhenoGraph defined a total of 19 different clusters as indicated. (**b**) Heatmap of the surface marker expression levels of cells within these 19 clusters with percentage of cells that are positive for each marker. Surface markers that are used to define the dominant T cell subset for each cluster are boxed. (**c**) Distribution of total CD4^+^ T cells, YFV-specific, EBV- specific, FLU B-specific and TT-specific T cells in UMAP.

Our staining panel allowed us to classify YFV-specific, TT-specific, FLU B HA-specific and EBV EBNA2-specific CD4^+^ T cells with respect to their expression of 21 distinct cell surface markers. Applying a classical biaxial analysis at the peak of the YFV specific T cell response (day 14), we observed that the phenotype of the recently primed YFV-specific T cells clearly differed from that of other specificities such as FLU B HA-specific T cells (data from a representative subject at day 14 is shown in Fig. S3). To more adequately analyze high dimensional cell surface phenotypes, taking into account various combinations of 21 surface markers, we applied a UMAP approach in conjunction with PhenoGraph^22^ to examine and compare the phenotypic features of EBV EBNA2, FLU B HA, TT and YFV tetramer positive cells across the seven time points included in our study. Both total CD4^+^ T cells and antigen-specific T cells were included in this analysis, allowing us to define an adequately comprehensive phenotypic landscape of these epitope specific T cells and total CD4^+^ T cells. This analysis revealed the presence of nineteen phenotype clusters, the majority of which could be clearly classified as either CD45RO^+^CD45RA^−^ memory (total of ten subsets) or CD45RO^−^CD45RA^+^ naïve (total of five subsets, including two CD45RA dim subsets) T cells (Fig. 3a,b and Fig. S4). Memory T cells could be further subdivided into distinct subsets that resembled expected T cell lineages: two CCR6^+^ CCR4^+^ Th17-like subsets (clusters 11, 12), a CXCR3^+^CCR4^−^ Th1-like subset (cluster 8), a CCR4^+^CXCR3^−^ Th2-like subset (cluster 18), a CD127^−^CD25^+^CD39^+^ Treg-like subset (cluster 2), and two cCXCR5^+^ subsets (clusters 10 and 17). In addition, there were three activated (CD38^+^) subsets within the memory population which were characterized by differential expression of ICOS, CD71 and CD69 (clusters 6, 7, and 9). All ten memory subsets were projected onto the upper portion of the UMAP while the five naïve subsets were projected onto the low portion of the UMAP. Four unconventional CD45RA^+^RO^+^ subsets were also observed (clusters 4, 5, 15, 16), but these represented less than 3% of the total CD4^+^ T cell populations (Table S3), possibly reflecting rare transitioning T cell states. As expected, most EBV EBNA2-specific, FLU B HA-specific, and TT-specific T cells and YFV-specific cells were situated within the various memory subsets located in the upper region of the UMAP projection. In addition, the data showed that T cells had unique phenotypic clusters based on their antigen specificity. These data generally suggested that CD4^+^ T cells that are targeted towards different microbes exhibit different phenotypes. In particular, TT-specific cells were commonly present within Th17-like phenotype clusters (clusters 11 and 12) and were distinct from the other three viral specificities (Fig. 3c). The clustering of YFV-specific cells was distinct from TT-specific, EBV EBNA2-specific and FLU B HA-specific T cells. Reflective of recent vaccination, a majority of YFV-specific cells were present within the activated T cell clusters (i.e. clusters 6, 7 and 9) and the PD1^+^cCXCR5^+^ cluster (cluster 10). Though a high percentage of both EBV EBNA2-specific and FLU B HA-specific cells were clustered in the Th1-like region (cluster 8), some EBV EBNA2-specific T cells were present within the PD1^+^ cCXCR5^+^ cluster (cluster 10). A summary of the different phenotypic clusters of these four distinct specificities of T cells is shown in Table S3. Unlike EBV EBNA2-specific, FLU B HA-specific, and TT-specific T cells, whose phenotypes were fairly static at different time points, YFV-specific cells at different time points clustered differently (Fig. S5A and Table S4). Greater than 75% of these YFV-specific cells between day 14 and the one year time points were within clusters 6–10 and 17, ie activated clusters, the TH1 cluster and the cCXCR5^+^ cluster. We did not observe appreciate development of YFV-specific cells in the TH2 or TH17 clusters (Table S4).

### UMAP and biaxial plot analysis of YFV-specific T cells show the presence of a cCXCR5^+^ subset

**Figure 4:**
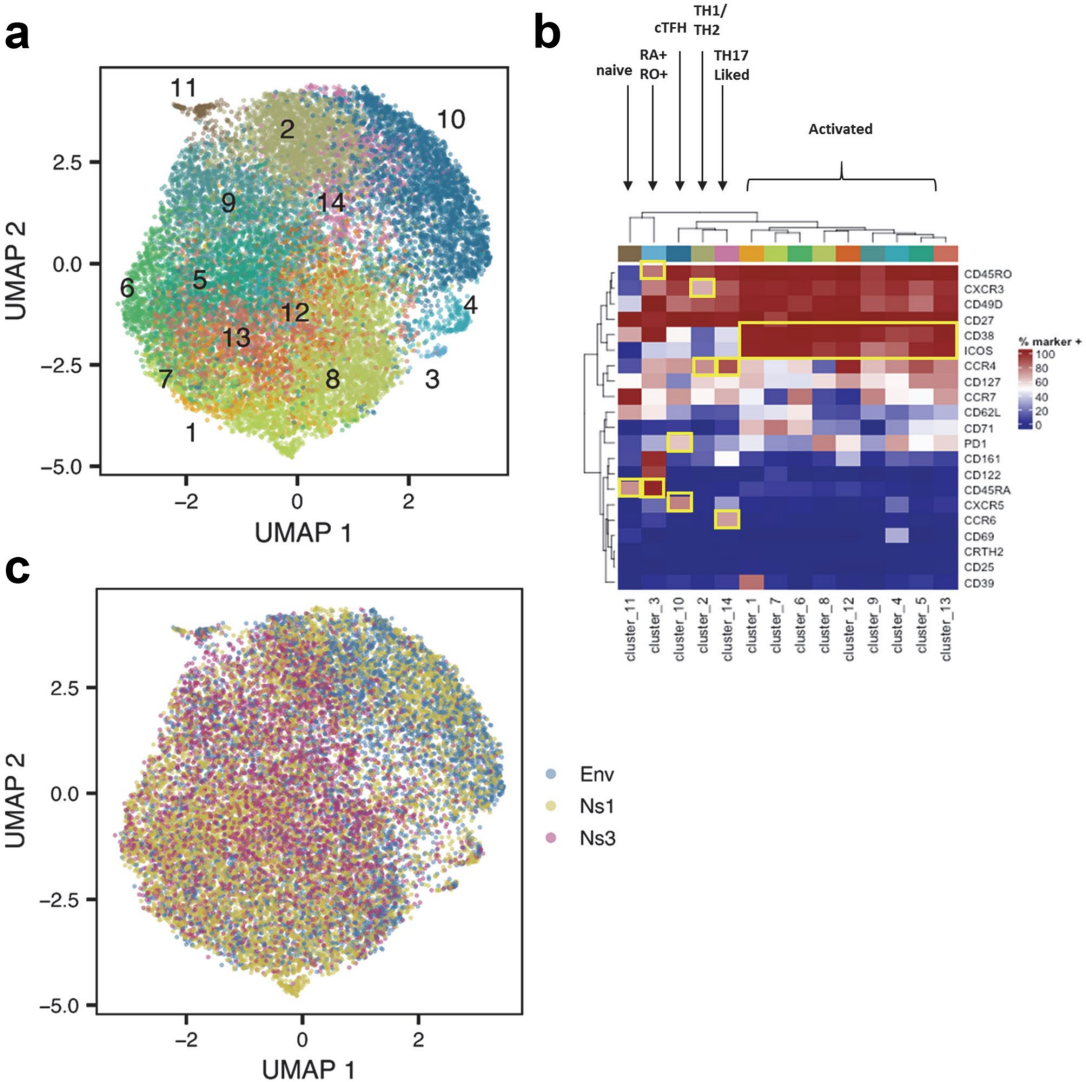
Cellular clustering of YFV epitope-specific CD4^+^ T cells pre and post YF-Vax vaccination. (**a**) UMAP and PhenoGraph analysis of surface marker expression of YFV ENV-specific, YFV NS1-specific, YFV NS3- specific CD4^+^ T cells for all 9 subjects at all time points (n = 58). A total of 5246 YFV ENV-specific, 8005 YFV NS1- specific and 4600 YFV NS3-specific cells as identified by YFV-specific tetramer staining were included in the analysis. PhenoGraph defined a total of 11 different clusters. (**b**) Heatmap of the surface marker expression level of cells within these 11 clusters with percentage of cells that were positive for each marker. Markers that are used to define T cell subsets or activation state are boxed. (**c**) Distribution of YFV ENV-specific, YFV NS1-specific and YFV NS3-specific CD4^+^ T cells in UMAP.

We next carried out a more focused analysis of YFV-specific CD4^+^ T cells, applying UMAP and PhenoGraph to specifically assess the phenotypic states of YFV tetramer positive T cells following vaccination (Fig. 4a,b). Greater than 98.5% of YFV specific cells were CD45RA^−^CD45RO^+^ memory cells (data not shown). The PhenoGraph clusters of most YFV specific T cells were marked by cells with expression of activation markers, including ICOS, CD38 (clusters 1, 4–9, 12 and 13). With the exception of cluster 9, all of these cells also expressed CD71 or PD1. Cells in cluster 1 upregulated CD39, a hallmark for Treg or chronically stimulated cells; cells in cluster 4 upregulated CD69, a very early activation marker. Most of the YFV cells in day 14 were located within the 9 clusters mentioned above (Fig. S5B). In contrast, cells in clusters 2, 10 and 14 lacked CD38 expression, most likely representing a transition in phenotype toward more differentiated cell states after initial activation. In particular, cells in cluster 10 expressed CXCR5 (Fig. 4a,b and Fig. S5C), which suggested that these were cCXCR5^+^ T cells. Surprisingly, YFV NS3-specific cells were largely absent in cluster 10 (Fig. 4c), indicating that most YFV NS3-specific T cells did not differentiate into cCXCR5^+^ T cells. YFV specific CD4^+^ T cells were also present in cluster 11 (naïve cells) and cluster 3 (CD45RA^+^CD45RO^+^) cells but these two clusters represented less than 1.5% of the total population.

**Figure 5:**
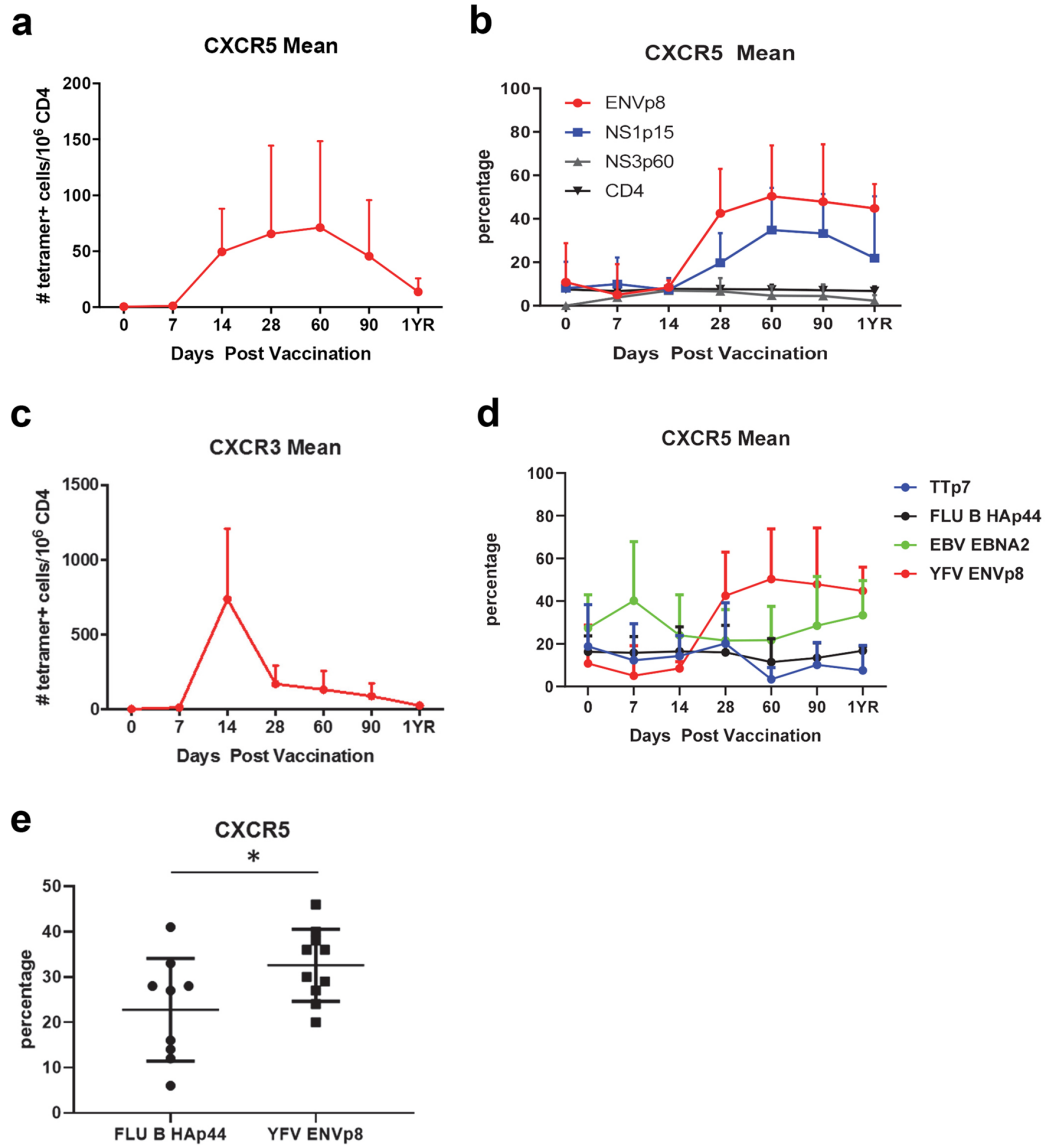
Kinetics of CXCR3^+^ and cCXCR5^+^CD4^+^ T cells post YF-Vax vaccination. (**a**) Average frequencies of YFV-specific cCXCR5^+^CD4^+^ at different time points post YF-Vax vaccination (average of summation of ENV, NS1, NS3 at each time point for each subject from a total of 9 subjects). (**b**) Percentage of total CD4^+^ T cells, YFV ENV-specific, YFV NS1-specific and YFV NS3-specific that were CXCR5^+^ at different time points post vaccination. (**c**) Frequencies of YFV-specific CXCR3^+^CD4^+^ at different time points post YF-Vax vaccination. (**d**) Percentage of YFV ENV-specific, TT-specific, EBV-specific and FLU B-specific CD4^+^ T cells that were CXCR5^+^ at different time points post vaccination. For a and c, n = 27 for the first 6 time point and n = 12 for the last time point. For b and d, n = 9 for each epitope for the first 6 time point and n = 4 for the last time point. (**e**) Percentage of CXCR5^+^ YFV ENV-specific cells (n = 10) at day 30 post YFV-Vax vaccination and percentage of FLU B HA-specific cells (n = 9) at day 30 post FLU vaccination. Shown are the means with SD. For e, unpaired 2 tailed Student’s t test was used, *indicates *p* value of < 0.05.

Biaxial analyses of YFV- specific cells show the appearance of cCXCR5^+^ T cells at day 14, and subsequent peak between day 30–60 (Fig. 5a and Fig. S6, S8). This analysis also indicated that a higher percentage of YFV ENV-specific and YFV NS1-specific CD4^+^ T cells were CXCR5^+^ in comparison to YFV NS3-specific T cells (Fig. 5b) with ENV-specific cells peaking at ~ 50% CXCR5 positivity 60 days after vaccination. Notably, the kinetics of cCXCR5^+^ T cells appearance was distinct from CXCR3^+^ T cells and total memory YFV-specific T cells (which peaked at day 14, followed by a rapid contraction, Figs. 2 and 5c) suggesting either a temporal difference in their induction or their homing and recirculation. Also of note, a higher percentage of YFV ENV-specific cells expressed CXCR5 in comparison to FLU B HA, TT and EBV EBNA2 specific T cells (Fig. 5d). This high percentage of CXCR5^+^ YFV ENV-specific T cells was maintained throughout the one year period of the study. Induction of CXCR5^+^ T cells might be expected to be merely a consequence of recent vaccination. However, the percentage of FLU B-specific T cells that expressed CXCR5 in subjects at day 30 post FLU vaccination was significantly lower than the percentage of YFV ENV-specific T cells that expressed CXCR5 in subjects at day 30 post YF-Vax vaccination (in this case by standard flow cytometry) (Fig. 5e).

### Ontogeny of circulatory CXCR5^+^ YFV-specific T cells

**Figure 6:**
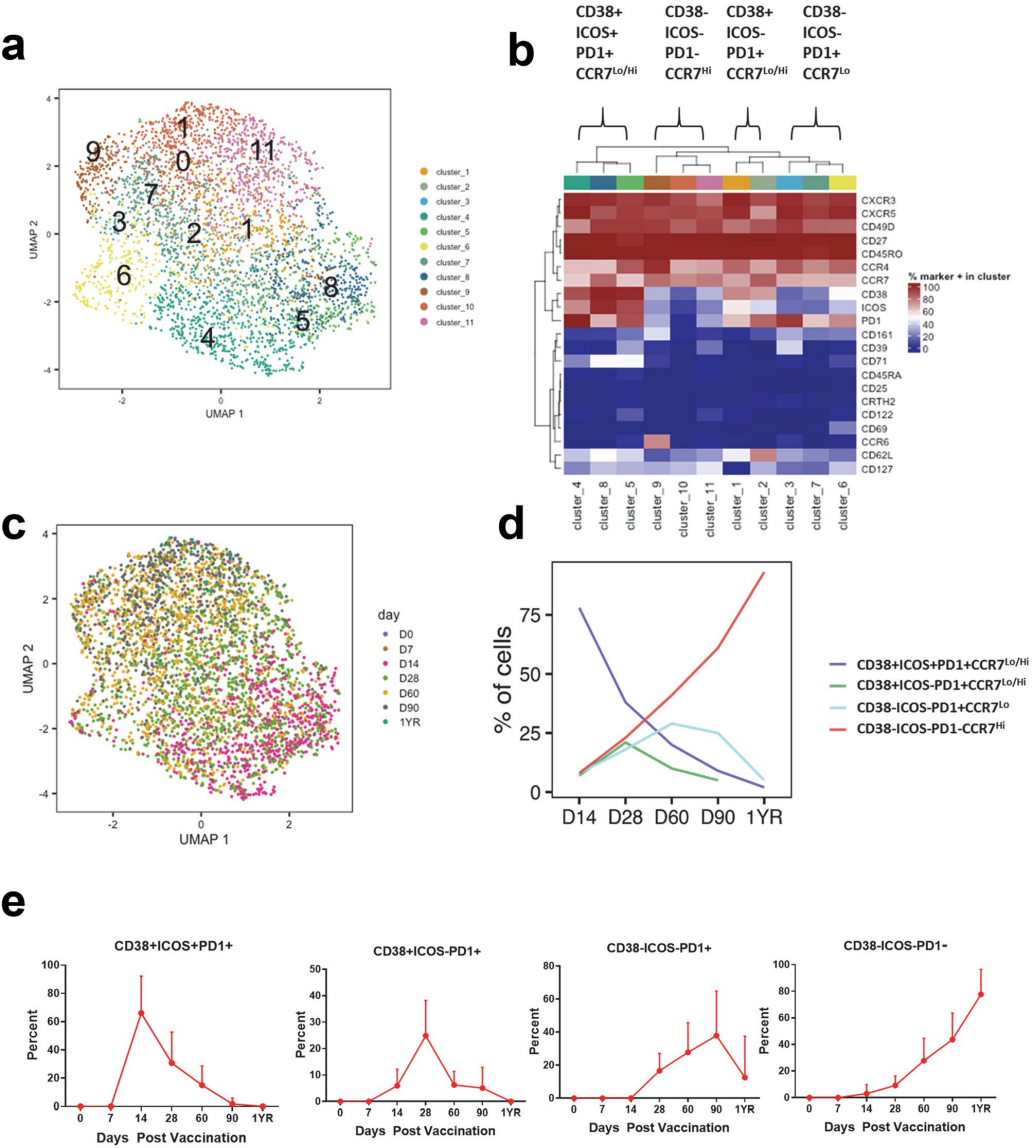
Cellular clustering of YFV-specific cCXCR5^+^ CD4^+^ T cells pre and post YF-Vax vaccination. (**a**) UMAP and PhenoGraph analysis of surface marker expression of YFV-specific cCXCR5^+^CD4^+^ T cells for all 9 subjects at all time points (n = 58). Only CXCR5^+^ YFV tetramer specific cells were included in the analysis. PhenoGraph defined a total of 11 different clusters. (**b**) Heatmap of hierarchical clustering of surface marker expression of these 11 clusters with percentage of cells that were positive for each marker. These 11 clusters were grouped by similarity into 4 different cCXCR5^+^ subsets. (**c**) Distribution of cCXCR5 + YFV -specific CD4^+^ T cells at different time point in UMAP. (**d**) Kinetics of the four different YFV-specific cCXCR5^+^CD4^+^ subsets as identified by UMAP and PhenoGraph. (**e**) Manual gating was used to identify different subsets of YFV ENV-specific cCXCR5^+^CD4^+^. Percentages of YFV ENV-specific cCXCR5^+^ T cells that expressed the indicated markers at different time points are as shown.

We further refined our understanding of the heterogeneity of the YFV-specific cCXCR5^+^ CD4^+^ T cells through additional UMAP and PhenoGraph analysis of the YFV-specific cCXCR5^+^ T cell subset. These data show that cCXCR5^+^ YFV-specific T cells can be classified into 11 distinct clusters. YFV specific cells with Tfr like characteristic were not detected. The 11 clusters can be further grouped into four subsets of closely related clusters based on their relative expression of CD38, ICOS, PD1 and CCR7 (Fig. 6a,b). These four subsets included a CD38^+^ICOS^+^PD1^+^CCR7^Lo/Hi^ subset (clusters 4, 8 and 5), a CD38^+^ICOS^−^PD1^+^CCR7^Lo/Hi^ subset (clusters 1 and 2), a CD38^−^ICOS^−^PD1^+^CCR7^Lo^ subset (clusters 3, 7 and 6) and a CD38^−^ICOS^−^PD1^−^CCR7^Hi^ subset (clusters 9, 10 and 11). The distributions of YFV specific cells between these cluster subsets varied at different time points after vaccination (shown in Fig. 6c). YFV-specific CXCR5^+^ cells at day 14 were mainly located in clusters 4, 8 and 5; whereas cells at day 90 and 1 year were mainly located in clusters 10 and 11. Time related changes in the percentage of YFV cells present in these different CXCR5 subsets as identified by UMAP and PhenoGraph are shown in Fig. 6d. The level of expression of PD1 of these four different subset overtime was also evaluated (Fig. S7).

These kinetics could be taken to suggest that shortly after vaccination CXCR5^+^ YFV specific cells with a CD38^+^ICOS^+^PD1^+^CCR7^Lo/Hi^ phenotype appear, but that these cells may then transition to become CD38^+^ICOS^−^PD1^+^CCR7^Lo/Hi^, CD38^−^ICOS^−^PD1^+^CCR7^Lo^, and finally CD38^−^ICOS^−^PD1^−^CCR7^Hi^. This interpretation is supported by the observation that level of PD1 expression is highest in the CD38^+^ICOS^+^PD1^+^CCR7^Lo/Hi^ subset, and the level of expression decreases overtime within the first 90 days (Fig. S7). To further assess this possibility of transition from CD38^+^ICOS^+^PD1^+^CCR7^Lo/Hi^ subset into CD38^−^ICOS^−^PD1^−^CCR7^Hi^ subset, we used manual gating to identify different subsets of cCXCR5 YFV-specific cells and performed a biaxial analyses of the eight different CXCR5^+^ subsets based on CD38, ICOS and PD1 for YFV-ENV cells at different time points (Fig. 6e and S8). Interestingly, CD38^+^ICOS^+^PD1^+^ cells first appeared at day 14, and their frequency peaked at day 28 (Fig. S8A). CD38^+^ICOS^−^PD1^+^, CD38^−^ICOS^−^PD1^+^ and CD38^−^ICOS^−^PD1^−^ subsets appeared later and peaked at day 28, day 60 and day 90 respectively (Fig. S8A). Of note, the CD38^−^ICOS^−^PD1^−^ subset is relatively absent in the first 28 days. The CD38^+^ICOS^+^PD1^−^, CD38^+^ICOS^−^PD1^−^, CD38^−^ICOS^+^PD1^+^ and CD38^−^ICOS^+^PD1^−^ subsets were minor subsets, with average frequencies of less than 2.5 per million CD4^+^ T cells at each time point (Fig. S8B). Examining time related changes in the percentages of T cells within each CXCR5^+^ subset at each time provided similar insights as observed earlier (Fig. 6e). On day 14, the majority of YFV-ENV CXCR5^+^ specific cells were CD38^+^ICOS^+^PD1^+^. The percentage of CD38^+^ICOS^−^PD1^+^ and CD38^−^ICOS^−^PD1^+^ peaked at day 28 and 90 respectively, while on day 360, more than 80% of the cells were CD38^−^ICOS^−^PD1^−^. Very similar kinetic of these different subsets were also observed for YFV NS1-specific CD4^+^ T cells (Fig. S8C). Therefore, both UMAP-PhenoGraph analysis and biaxial plot of manually gated analysis results support the idea that cCXCR5^+^ T cells transition from a PD1^+^ICOS^+^CD38^+^CCR7^Lo^ to a PD1^−^ICOS^−^CD38^−^CCR7^Hi^ phenotype following YF-Vax vaccination.

## Discussion

We used metal labeled class II tetramer reagents and mass cytometry to examine YFV specific, FLU B HA-specific, EBV EBNA-specific and TT-specific CD4^+^ T cells in healthy subjects after YF-Vax vaccination. As expected, primary YFV vaccination only elicited measurable increases in the frequencies of YFV-specific cells. Consistent with prior studies, there was no detectable bystander expansion of CD4^+^ T cells specific for other viruses^23^. YF-Vax elicited a high proportion of YFV-specific cCXCR5^+^ T cells, such that nearly 50% of CD4^+^ YFV ENV-reactive T cells were CXCR5^+^ 60 days after vaccination. Furthermore, elicitation of YFV-specific cCXCR5^+^ cells was durable in that close to 45% of YFV ENV-reactive T cells retained CXCR5 expression one year after vaccination. It is known that VF-Vax is highly effective, generating high titer, high affinity antibodies that confer lifelong protection against YFV^17^. Indeed, our work demonstrated that YF-Vax vaccination elicited a significantly higher percentage of cCXCR5^+^ T cells than influenza vaccination. The observation that YF-Vax promotes the emergence of high frequencies of YFV-specific cCXCR5^+^ T cells suggests that this peripheral T cell population may indeed parallel the presence and activity of YFV-specific T_FH_ in GC and secondary lymphoid organs.

The kinetics of the expansion that we observed for cCXCR5 YFV-specific CD4^+^ T cells differs from previous studies, in which the frequencies of antigen specific cCXCR5^+^ T cells peaked around day 7^9,11,12^. The current study was carried out in the setting of primary vaccination whereas in earlier studies the setting was secondary vaccination in which pre-existing memory cCXCR5^+^ T cells should already be present. This delay in the appearance of cCXCR5^+^ T cells after primary vaccination implies naïve T cells must be activated and undergo a differentiation process, which may include a period of retention in secondary lymphoid tissues before emerging as cCXCR5^+^ T cells in the periphery.

Priming of naïve T cells with antigen-loaded DC was shown to be sufficient to induce CXCR5 expression. It is also well established that the interaction of T cells with B cells is essential for the maintenance of CXCR5 expression^24–26^. Induction of CXCR5 on the surface of recently primed naïve T cells would be expected to direct these cells into a follicular area which has high level of CXCL-13, hindering their exit from lymph nodes and delaying the appearance of cCXCR5^+^ T cells in the periphery^27^. If cCXCR5^+^ cells do indeed originate from secondary lymphoid tissues, loss of expression of Bcl-6 and decreased levels of CXCR5 would be expected consequences of lack of contact with B cells following their re-circulation into peripheral blood^24–26,28,29^.

Having established that cCXCR5^+^ T cells comprise a significant component of the T cell response elicited by YFV vaccination, we applied both an unbiased analytical approach and a standard gating strategy to draw insights about their ontogeny. We observed that YFV specific cCXCR5^+^ T cells existed in multiple phenotypic clusters. One key population was mainly ICOS^+^PD1^+^CD38^+^ with modest expression of CCR7. This population most resembled GC-T_FH_ based on surface marker expression and exhibited delayed accumulation in the periphery, implying that these T cells could be emigrants from GC-T_FH_. Based on the relative kinetics of their emergence following vaccination, we speculate that these triple positive cCXCR5^+^ cells transition to become CD38^+^ICOS^−^PD1^+^ and then CD38^−^ICOS^−^PD1^+^ before accumulating in the periphery as CD38^−^ICOS^−^PD1^−^CCR7^+^ cells. In particular, the relative absence of CD38^−^ICOS^−^PD1^−^CXCR5^+^ T cells in the first 28 days post vaccination suggests that majority of triple negative antigen-specific cCXCR5^+^ T cells are derived from other populations of PD1^+^ cCXCR5^+^ T cells. Given the observations that PD1^+^cCXCR5^+^ and CD38^+^ICOS^+^ cCXCR5^+^ subsets have gene expression profile that resemble GC-T_FH_^8,14^, it is plausible that all these different subsets of YFV specific cCXCR5^+^ T cells, including the CD38^−^ICOS^−^PD1^−^CXCR5^+^ T cells are descendants of pre-T_FH_ and GC-T_FH_.

A recent study that examined CD4^+^ T cell within the lymph nodes of HIV-infected subject suggested that ICOS^+^PD1^+^CXCR5^−^ cells are derived from ICOS^+^PD1^+^CXCR5^+^ T cells^30^. As the current study focused on CXCR5^+^ T cells within periphery blood after vaccination, we cannot rule out the relationship between these two populations within the lymph node. On the other hand, the kinetics of induction of ICOS^+^PD1^+^CXCR5^−^ and ICOS^+^PD1^+^CXCR5^+^ in the peripheral circulation show that these 2 populations peaked around day 14 and day 28 respectively (Figs. S8A,D), implicating that in the settings of vaccination and acute infection, the majority of antigen specific ICOS^+^PD1^+^CXCR5^−^ cells in peripheral blood in the acute phase were simply activated T cells and not derived from the cCXCR5^+^ population.

Our results indicated that there is an apparent hierarchy in the induction of cCXCR5^+^ T cells for different YFV antigens: a higher percentage of YFV-ENV-specific cells expressed CXCR5 compared to YFV NS1-specific cells, and very few NS3-specific cells expressed CXCR5. Earlier studies concluded that TCR avidity for pMHC dictates the differentiation of T_FH_^31^. Alternatively, differences in the relative abundance of YFV- specific B cells of different antigen specificities could also play a role, since cognate interactions between B cells and T cells are required to maintain expression of CXCR5^24–26^. Given that ENV is a viral surface antigen and NS1 as a membrane bound protein of infected cell^32^, whereas NS3 is a helicase/protease that is present internally^33^; the relatively poor induction of NS3-specific cCXCR5^+^ T cells could be explained by a corresponding disparity in the generation of NS-3 specific B cells. Indeed, it was previously reported in the setting of influenza vaccination that viral surface proteins elicit more cCXCR5^+^ T cells than internal proteins^34^. In any case, observed differences in CXCR5 expression (and that of other surface markers) amongst EBV, influenza, TT and YFV-specific CD4^+^ T cells can be taken to suggest that factors such as the recency, route of exposure, nature of the antigen, and affinity of the epitope for MHC can profoundly influence CD4^+^ T cell differentiation and phenotype.

In conclusion, our study highlighted the ontogeny of different subsets of antigen specific cCXCR5^+^ T cells. The delayed appearance of CXCR5^+^ICOS^+^PD1^+^ CD38^+^ YFV-specific cells in the periphery in comparison to the CXCR3^+^CXCR5^−^ subset implies that the antigen specific CXCR5^+^ICOS^+^PD1^+^ CD38^+^ cells are being preferentially detained within the secondary lymphoid tissues for pre-T_FH_ or T_FH_ activities. We also demonstrated the transition of CXCR5^+^ICOS^+^PD1^+^CD38^+^ YFV-specific cells through the ICOS^−^PD1^+^CD38^+^ and ICOS^−^PD1^+^CD38^+^ intermediate states into the CXCR5^+^ICOS^−^PD1^−^CD38^−^ subset. Together, these results imply that most antigen-specific cCXCR5^+^ T cells are derived from pre-T_FH_, and/or T_FH_ and may have the potential to gain pre-T_FH_ and T_FH_ function upon antigen specific rechallenge.

## Methods

### Human subjects

Blood samples were obtained from nine DRB1*0301 subjects enrolled in the YF-Vax longitudinal study. Samples were collected just prior to YF-Vax vaccination and 7, 14, 28, 60, 90 and 360 days post vaccination. Sample size was selected from preliminary data that indicated nine subjects would be sufficient to observe an increase of 50 tetramer positive, CXCR5 cells per million CD4 T cells at day 28 relative to day 0 with 80% power at the 5% significance level. Samples were obtained from an additional ten subjects 30 days after YF-Vax vaccination for a cross sectional study. The HLA of this group included DRB1*0301, DRB1*1101 and DRB1*1501. All subjects that were recruited to receive the YF-Vax vaccine had no prior history of YF-Vax vaccination. A total of nine subjects with either HLA-DRB1*1101 or DRB1*1501 that had received the influenza vaccine 30 days earlier were also recruited. The study was approved by Benaroya Research Institute IRB, and all subjects were recruited with informed consent.

### Antibody labeling

Maxpar Antibody Labeling Kits containing the desired metals were purchased from Fluidigm (San Francisco, CA) and purified antibodies were purchased from indicated vendors (Table S2). Labeling of antibody was performed according to manufacturer’s specifications. Briefly, polymer was resuspended in 95 μL L-buffer along with 5 μL desired lanthanide metal and incubated at 37 °C for 35 min. Meanwhile, the desired antibody was reduced by addition of 100 μL R-buffer containing TCEP followed by incubation at 37 °C for 25 min. After incubation, the lanthanide loaded polymer was purified by washing and resuspending the sample in C-buffer. The lanthanide loaded polymer in C-buffer was then mixed with the partially reduced antibody and incubated at 37 °C for 2 h. After incubation, lanthanide labeled antibody was washed 4 times with W-buffer and resuspended in antibody stabilization buffer for use in staining.

### pMHC II tetramer production

Myc-tagged DRA1/DRB1*0301 HLA class II and untagged DRA1/DRB1*0301, DRA1/DRB1*1101 and DRA1/DRB1*1501 monomer reagents were generated essentially as previously described^29,35^. In brief, transfected S2 cells were expanded to a 2  L volume in spinner flasks (Bellco, Vineland NJ) and induced for 5 days with 1 mM copper sulfate, adding 2 μg mL^−1^ biotin to ensure efficient protein biotinlyation. Supernatants were separated from intact cells by centrifugation (11,000*g*), separated from debris using a 0.2 μm filter (ThermoFisher, Waltham, MA) and then affinity purified using L243 coupled with CNBr-Activated Sepharose 4B (GE Healthcare, Pittsburgh, PA). Class II protein was eluted at pH 11.5, equilibrated using pH 4.0 Tris buffer and exchanged into a pH 6.0 storage buffer (0.2 M sodium phosphate). Class II monomers were loaded with individual peptides by incubating for 72 h at 37°°C in the presence of 2.5 mg mL^−1^ *n*-octyl-β-d-glucopyranoside (Sigma, St Louis, MO). Tetramers were formed by individually incubating class II molecules with metal labeled streptavidin or PE-labeled streptavidin (Thermo Fisher Scientific) for 6–18 h at room temperature at a molar ratio of 8:1. Metal labeled streptavidin were produced as described earlier^36,37^.

### Mass cytometry staining and data acquisition

PBMCs were isolated from donors’ whole blood by ficoll density gradient separation on indicated days after YFV vaccination. Approximately twenty million PBMCs were taken for tetramer staining and resuspended in 200 μL TCM (RPMI, 10% human serum, l-glutamine, HEPES buffer, sodium pyruvate). Dasatinib (Santa Cruz Biotechnology) was then spiked in to the resuspended cells at a final concentration of 50 nM, and the cells were placed at 37 °C for 5 min. HLA DRB1 *0301 myc-labeled monomer loaded with indicated peptides (Table S1) were conjugated with appropriate streptavidin labeled lanthanides metals (provided by Evan Newell). After incubation with 50 nM dasatinib for 5 min at 37 °C, 3 μL of each tetramer (labeled with indicated lanthanides) were added to PBMCs in 200 μL TCM. Cells were incubated at room temperature for 2 h, with brief mixing every half hour. After 2 h, cells were washed with 3 mL run buffer (1X PBS, EDTA, sodium azide). Cell pellets were resuspended in 175 μL run buffer and 25 μL anti-c-myc microbeads (Miltenyi Biotec, Germany) were added to cells and incubated for 15 min at 4 °C. Cells were then washed with 3 mL run buffer and resuspended in 1 mL run buffer and placed over a pre-wet MS column (Miltenyi Biotec, Germany) on magnet. Twenty microliters of sample were taken for pre-enrichment analysis. MS column was washed with 1 mL run buffer, then taken off magnet and eluted with 1 mL run buffer. Enriched cells along with pre-enrichment sample were stained with extracellular antibody cocktail for 25 min in the dark at RT. During the last minute of antibody incubation cisplatin was spiked into sample, and subsequently quenched with 3 mL run buffer. Cells were then washed with 1XPBS and resuspended in 500 μL 1XPBS with iridium and were incubated for 30 min at RT. Cells were washed with 1XPBS, spun down and resuspended in 150 μL IC fixation (eBiosciences) buffer and incubated at RT for 45 min. Cells were then washed with 1XPBS, followed by 2 washes in ddiH_2_O. Cells were resuspended in ddiH_2_O, and EQbeads (Fluidigm, San Francisco, CA) were spiked into samples. Cells were immediately run on the CyTOF. Files were normalized using EQbeads and CyTOF 2 software and FCS files were then analyzed on FlowJo software. For tetramer analysis, the total cells positive for each of the tetramer designated lanthanides were gated individually. Subsequently, using the Boolean gating function in Flowjo, each tetramer population was gated so that all cells specific for an individual epitope stained positive for both of the tetramer-lanthanides designated for that epitope, but stained negative for the remaining lanthanide labeled tetramer designated for all other epitopes. Frequency of epitope specific cells were calculated by using the formula: F = (1,000,000 × tetramer positive events from enriched tube)/(10 × number of CD4^+^ T-cells from the ‘Pre’ fraction).

### Mass cyotmetry data analysis

Mean and standard deviations of frequency data and percent positive data were computed to summarize gating results for both individual markers and cell subsets. FCS files were analyzed using custom R scripts. Files were read into the R using the flowCore package^38^. Marker intensities were transformed using an inverse hyperbolic sine transformation (arcsinh) using parameters a = 0, b = 0.2. Dimensionality reduction was performed using the Uniform Manifold Approximation and Projection (UMAP) algorithm as implemented in the umap R package^22^. To adjust for batch differences between samples, a z-score normalization was applied to each marker relative to the total CD4 T cell expression for that marker on the same subject and visit prior to dimensionality reduction using UMAP.

Flow data were clustered using the PhenoGraph algorithm as implemented in the R Phenograph package^39^. Cluster membership was visualized using the ComplexHeatmap package according to the percent of cells within a cluster that were considered positive for that biomarker in a previous biaxial gating analysis^40^. Hierarchical clustering was generated using a Euclidean distance metric and complete agglomeration method.

### Tetramer and antibody reagents for standard flow cytometry.

PE-conjugated DRB1*0301/YFV ENV43-59, DRB1*1101/YFV373-389, DRB1*1501/YFV ENV457-473 tetramers were used for examining YFV ENV-specific cells. PE-conjugated DR1101/FLU HA161-189 and DR1501/FLU HA433-452 were used for examining FLU HA specific CD4^+^ T cells. Staining protocol is similar to those of metal tagged tetramers. Cells were pretreated with dasatinib and were then stained with PE-tetramers at RT for 120 min. Cells were then incubated with anti-PE beads at 4 °C for 20 min. PE-tetramer positive cells were enriched through a magnetic column. Cells were than stained with anti-CD4-APC-H7, anti-CD45RA-v500, anti-CXCR5-APC, anti-CD14-PerCP, anti-CD19-PerCP and Via-probe (all BD Biosciences) for 20 min at 4 °C, washed and then analyzed on a BD LSRII.

## Supplementary information

Supplementary file1

## Data Availability

Data is publicly available at https://github.com/BenaroyaResearch/YFV-CyTOF-vaccine-study.

## Code availability

Analysis code is publicly available at https://github.com/BenaroyaResearch/YFV-CyTOF-vaccine-study.
