# Supplementary material for: Ontogeny of different subsets of yellow fever virus-specific circulatory CXCR5+ CD4+ T cells after yellow fever vaccination
Source: Sci Rep. 2020 Sep 24;10:15686. doi: 10.1038/s41598-020-72610-6 (PMC7519049; doi:10.1038/s41598-020-72610-6)
Supplement: Supplementary file 1 — Supplementary file1 [file 41598_2020_72610_MOESM1_ESM.pdf]

Supplemental Material

Ontogeny of different subsets of yellow fever virus-specific circulatory CXCR5$^+$ CD4$^+$ T cells after yellow fever vaccination

Quinn DeGottardi[1,2], Theresa J Gates[1], Junbao Yang[1,3], Eddie A James[1], Uma Malhotra[1,4], I-Ting Chow[1], Yannick Simoni[5,], Michael Fehlings[6], Evan W Newell[5], Hannah A. DeBerg[1], William W Kwok[1,7,*]

1 Benaroya Research Institute at Virginia Mason Medical Center, Seattle, WA

2 Current address, Adaptive Biotechnologies, Seattle, WA

3 Current address, Cs-Bay Therapeutics, Newark, CA

4 Virginia Mason Hospital, Seattle, WA

5 Vaccine and Infectious Disease Division, Fred Hutchinson Cancer Research Center, Seattle, WA

6 Singapore Immunology Network, Agency for Science Research and Technology, Singapore

7 Department of Medicine, University of Washington, Seattle, WA

* Corresponding Author: William W Kwok, Benaroya Research Institute at Virginia Mason, 1201, 9th Ave, Seattle, WA 98101, bkwok@benaroyaresearch.org

Figure Legends

Figure S1. Gating Strategy in identifying and defining the surface phenotype of antigen specific $CD4^+$ T cells. For identifying epitope specific cells, singlet live cells that were $CD45^+CD14^-CD19^-CD3^+CD4^+$ were manually gated. Boolean gating was then applied to identify cells that were labeled with the two specific metals which were associated with the epitope of interest, and negative for the other 5 irrelevant tetramer metals. This double positive population was then gated to analyze the surface markers of interest. The figure also depicted the overlaid of these epitope specific cells onto other CD4+ T cells that were outside the Boolean gate.

Figure S2. Expression of CD38 on surface of Flu B HA-specific cells from subject YFV027. PBMCs from subject YFV027 at day 0, 14 and 60 were stained with a panel of 6 different DRB1*0301 tetramers and 28 different antibodies as listed in Table S1 and S2. DRB1*0301 restricted FLU B HA-specific cells were identified by Boolean gating strategy and overlaid onto total CD4+ T cells. Surface expression of CD38 on these cells are as shown.

Figure S3. Differences in cell surface phenotype of YFV NS1-specific and FLU B HA-specific $CD4^+$ T cells at day 14 post YF-Vax vaccination. PBMCs from a DRB1*0301 subject were stained with a panel of 6 different DRB1*0301 tetramers and 28 different antibodies as listed in Table S1 and S2. YFV NS1-specific and FLU B HA-specific cells were identified by Boolean gating strategy. Surface markers that are distinct between YFV NS1-specific and FLU B HA-specific $CD4^+$ are marked by arrows.

Figure S4. Expression patterns of surface markers of total $CD4^+$ T cells and antigen specific cells as visualized by UMAP. UMAP was applied for visualization of the high dimensional CyTOF data set which examines the surface expression of 21 different markers in total $CD4^+$ T cells, YFV-specific, EBV-specific, FLU-specific and TT-specific $CD4^+$ T cells. UMAP projection of a total of 82,811 $CD4^+$ T cells from all 9 subjects at all time points assayed

(a total of 58 samples), including 17,851 YFV-specific, 1,280 EBV-specific, 2,913 FLU-specific, 2,767 TT-specific $CD4^+$ T cells and from 58,000 non-YFV, non-EBV, non-FLU, and non-TT $CD4^+$ T cells with 1,000 cells from each subject at each time point. The expression level of each indicated marker is as shown.

Figure S5. Distribution of YFV-specific cells in UMAP pre and post YF-Vax vaccination highlighted by different visit. (A) UMAP was applied for visualization of the high dimensional CyTOF data set which examines the surface expression of 21 different markers in total $CD4^+$ T cells, YFV-specific, EBV-specific, FLU-specific and TT-specific $CD4^+$ T cells for all 9 samples at all time points as shown in Fig. 4 and S3. The distributions of YFV-specific $CD4^+$ T cells at different time point pre and post YF-Vax vaccination are as shown. Both non-YFV- specific CD4+ T cells and YFV-specific are included in the analysis. **(B)** UMAP was applied for visualization of the high dimensional CyTOF data set which examine the surface expression of 21 different markers in YFV ENV-specific, YFV NS1-specific, YFV NS3-specific $CD4^+$ T cells for all 9 samples at all time points as shown in Fig. 4. A total of 5,246 YFV ENV-specific, 8005 YFV NS1-specific and 4,600 YFV NS3-specific cells as identified by YFV-specific tetramer staining were included in the analysis. Non-YFV-specific CD4+ T cells were excluded in the analysis. The distributions of YFV-specific $CD4^+$ T cells at different time point pre and post YF-Vax vaccination are as shown. **(C)** Expression of CXCR5 in YFV-specific cells as projected by UMAP.

Figure S6. CXCR5 expression of YFV ENV-specific CD4+ T cells at different time point post vaccination. PBMCs from a DRB1*0301 subject were stained with a panel of tetramers and antibodies at day 14, 28 and 60 post vaccination. YFV-ENV specific T cells were identified by using Boolean gates. The percentages of YFV ENV-specific cells that expressed CXCR5 are shown. Frequencies of these YFV ENV-specific $CXCR5^+$ cells were determined to be at 39, 66 and 111 per million $CD4^+$ cells at day 14, 28 and 60 respectively.

Figure S7. Relative expression of PD1 of different subsets of cCXCR5 YFV-specific cells overtime. Expression of PD1 on the 4 groups of cCXCR5 T cells by visit. The y-axis shows the resutlf of averaging the normalized marker intensity values over all subjects.

Figure S8. Frequencies of different subsets YFV- specific cCXCR5^{+}CD4^{+} T cells at different time points post vaccination. (A) Average frequencies of CD38^{+}ICOS^{+}PD1^{+}, CD38^{+}ICOS^{-}PD1^{+}, CD38^{-}ICOS^{-}PD1^{+} and CD38^{-}ICOS^{-}PD1^{-} YFV ENV-specific cells at different time points post vaccination. **(B)** Average frequencies of CD38^{+}ICOS^{-}PD1^{-}, CD38^{-}ICOS^{+}PD1^{-}, CD38^{-}ICOS^{+}PD1^{+} and CD38^{+}ICOS^{+}PD1^{-} YFV ENV-specific cells at different time points post vaccination. **(C)** Percentages of YFV NS1- and ENV- specific cCXCR5^{+} cells that expressed the indicated markers at different time points are as shown. **(D)** Average frequencies of ICOS^{+}PD1^{+}CXCR5^{-} YFV-ENV specific CD4^{+} T cells at different time points post vaccination. Shown are the means with SD (n=9 for the first 6 time point, and n = 4 for the last time point).

Figure S1

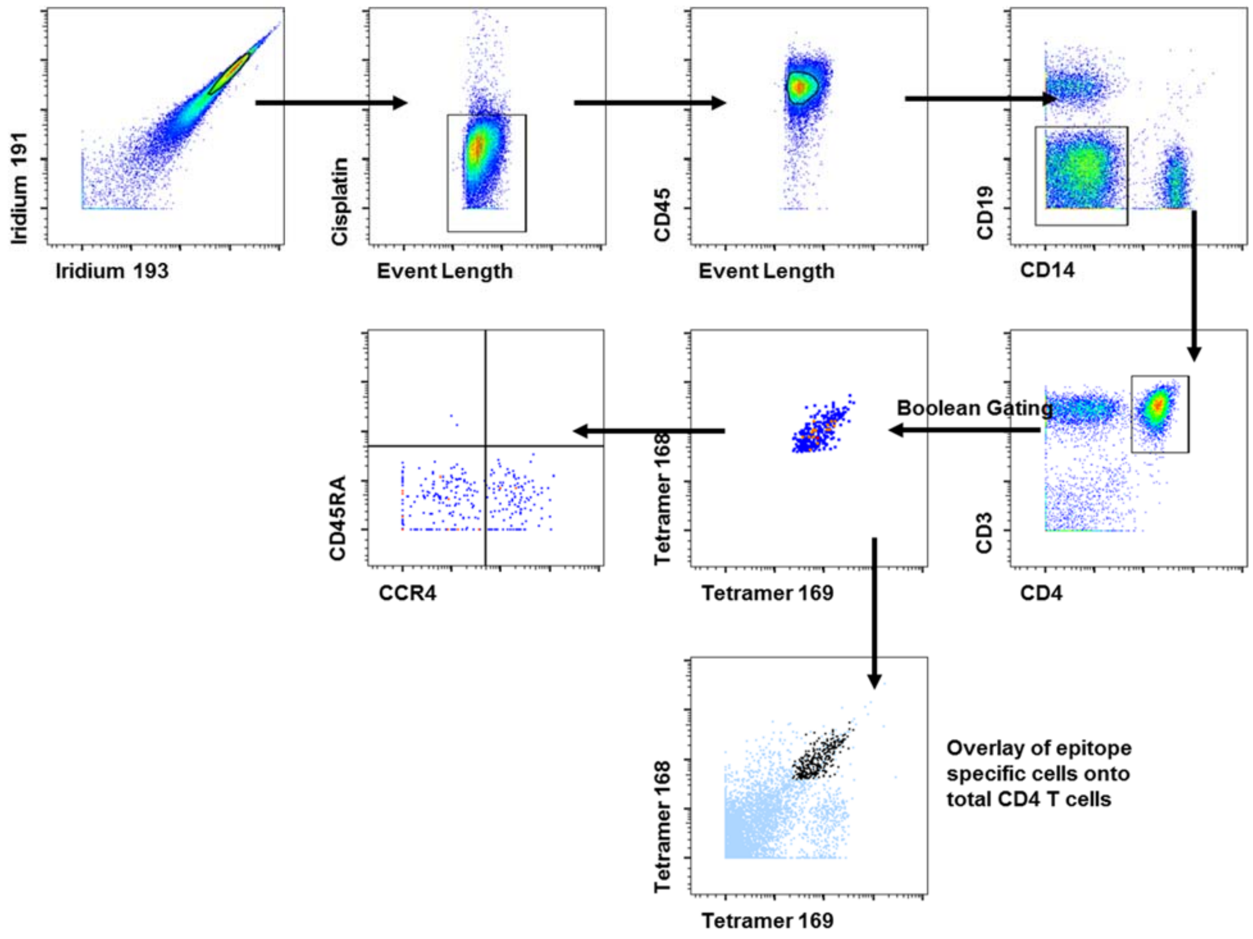
Iridium 191
Iridium 193
Cisplatin
Event Length
CD45
Event Length
CD19
CD14
CD45RA
CCR4
Tetramer 168
Tetramer 169
Boolean Gating
CD3
CD4
Tetramer 168
Tetramer 169
Overlay of epitope specific cells onto total CD4 T cells

Figure S2

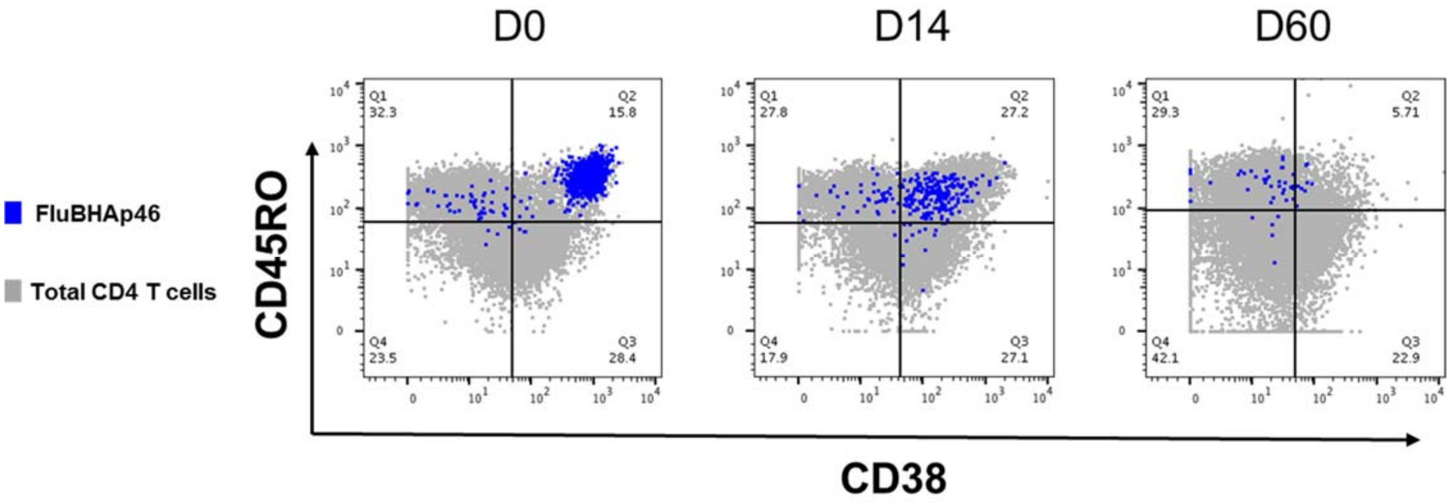

FluBHAp46
Total CD4 T cells
D0
D14
D60
CD45RO
CD38
Q1
32.3
Q2
15.8
Q4
23.5
Q3
28.4
Q1
27.8
Q2
27.2
Q4
17.9
Q3
27.1
Q1
29.3
Q2
5.71
Q4
42.1
Q3
22.9

Figure S3

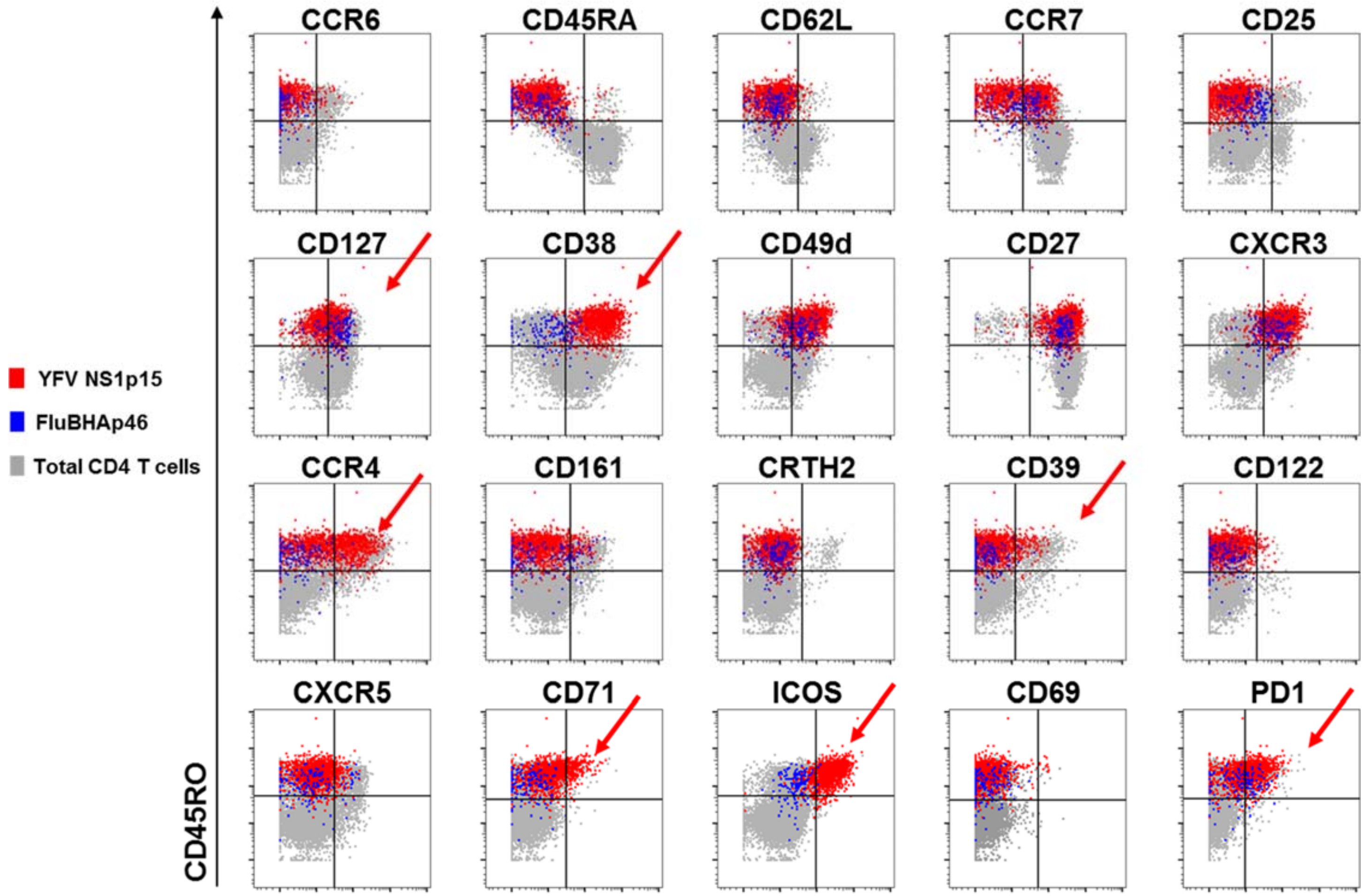

YFV NS1p15
FluBHAp46
Total CD4 T cells
CD45RO
CCR6
CD45RA
CD62L
CCR7
CD25
CD127
CD38
CD49d
CD27
CXCR3
CCR4
CD161
CRTH2
CD39
CD122
CXCR5
CD71
ICOS
CD69
PD1

Figure S4

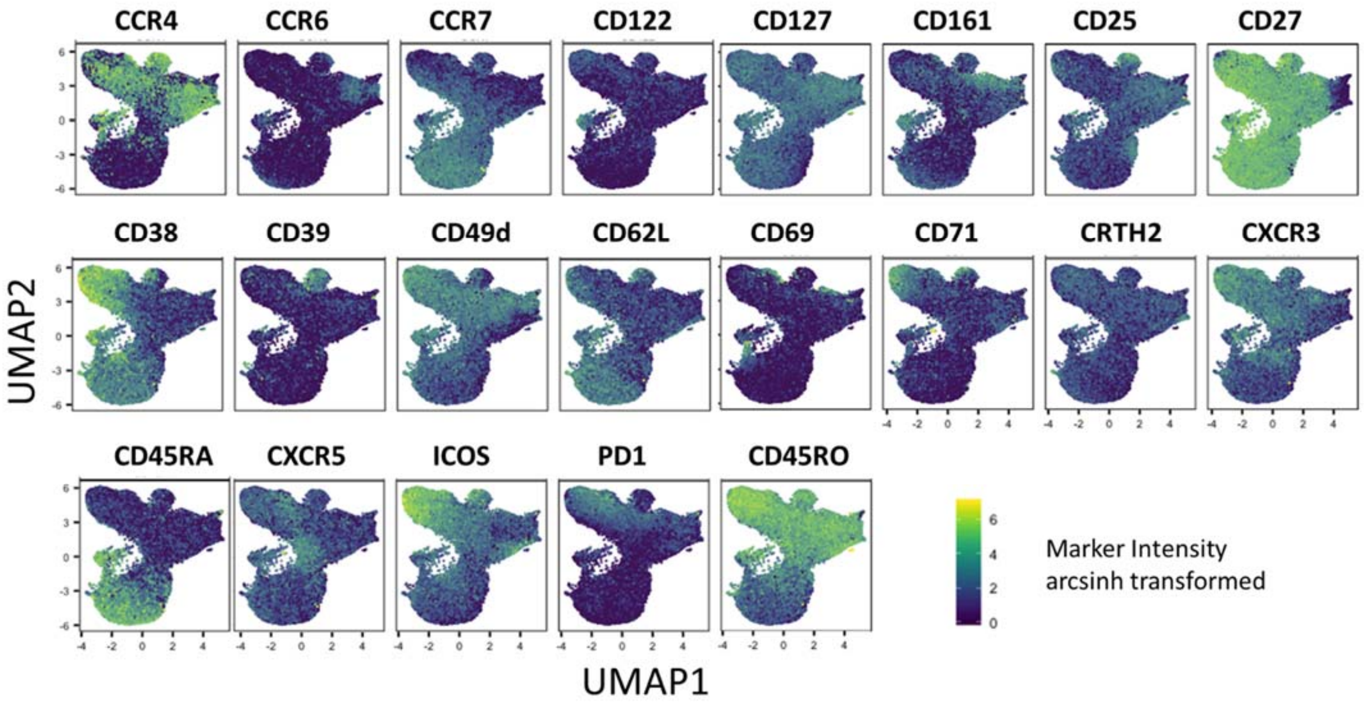
CCR4
CCR6
CCR7
CD122
CD127
CD161
CD25
CD27
CD38
CD39
CD49d
CD62L
CD69
CD71
CRTH2
CXCR3
CD45RA
CXCR5
ICOS
PD1
CD45RO
UMAP2
UMAP1
Marker Intensity
arcsinh transformed

Figure S5

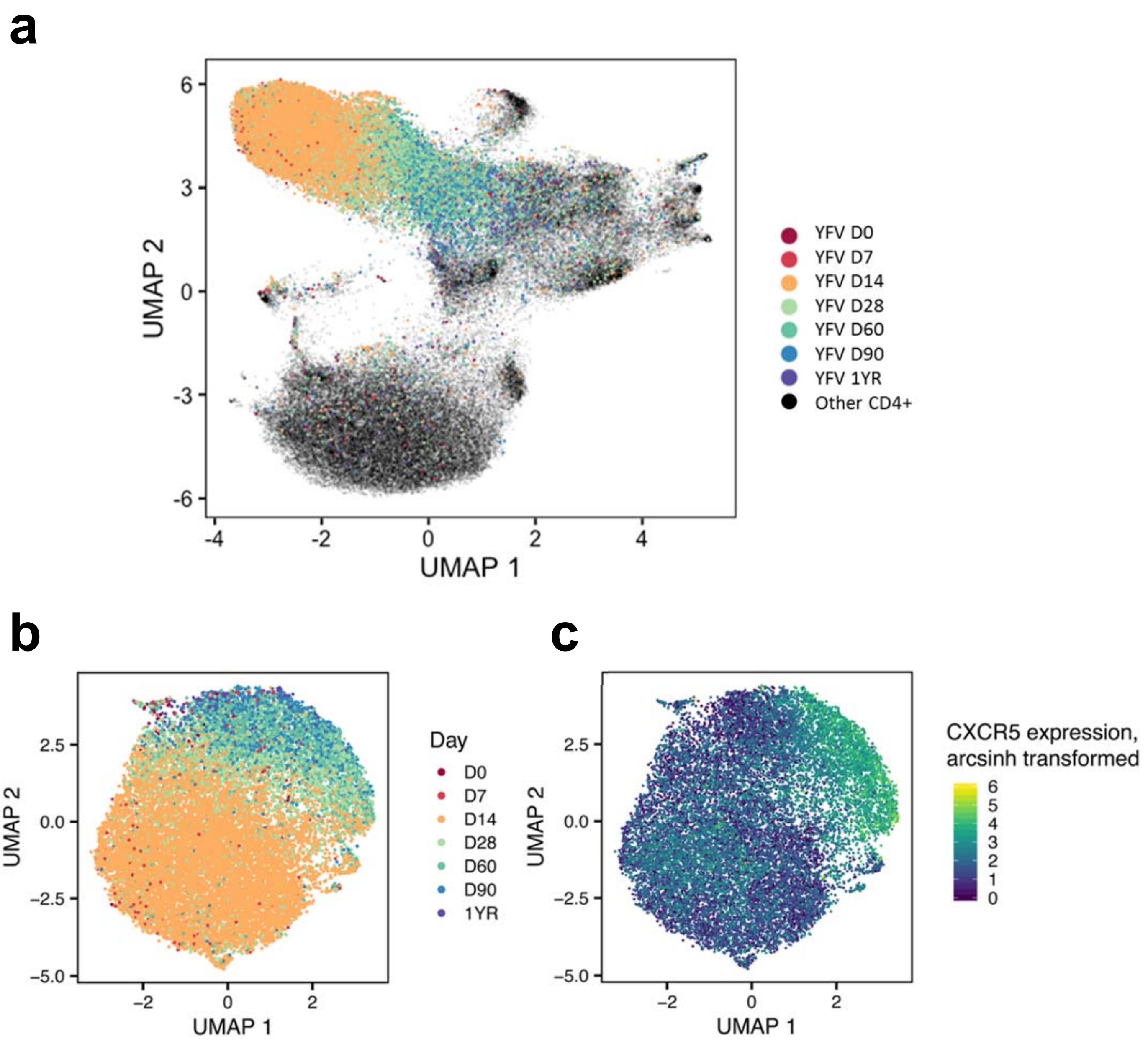
a
b
c
UMAP 1
UMAP 2
YFV D0
YFV D7
YFV D14
YFV D28
YFV D60
YFV D90
YFV 1YR
Other CD4+
Day
D0
D7
D14
D28
D60
D90
1YR
CXCR5 expression,
arcsinh transformed

Figure S6

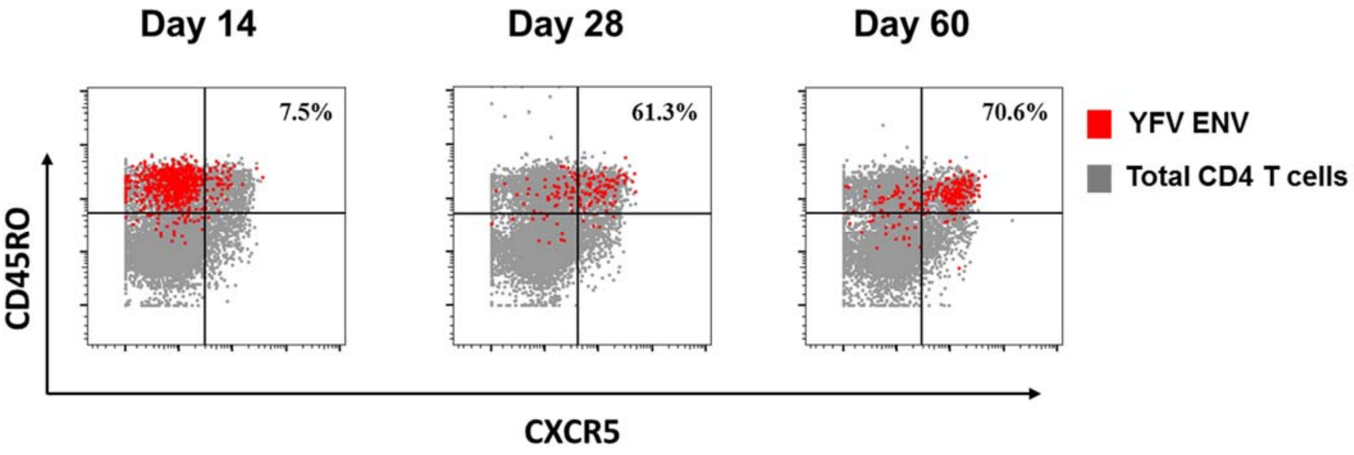
Day 14
Day 28
Day 60
7.5%
61.3%
70.6%
YFV ENV
Total CD4 T cells
CD45RO
CXCR5

Figure S7

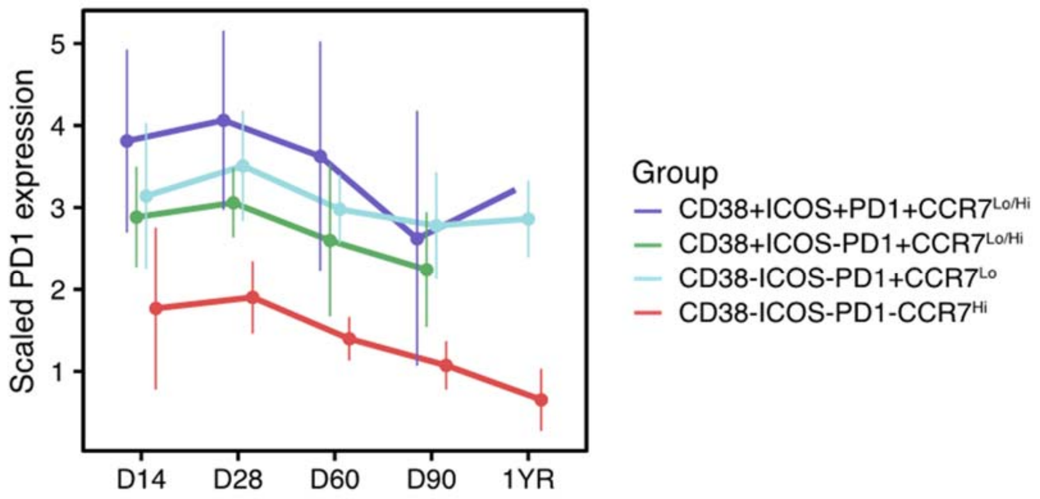

Scaled PD1 expression
5
4
3
2
1
D14
D28
D60
D90
1YR
Group
CD38+ICOS+PD1+CCR7Lo/Hi
CD38+ICOS-PD1+CCR7Lo/Hi
CD38-ICOS-PD1+CCR7Lo
CD38-ICOS-PD1-CCR7Hi

Figure S8

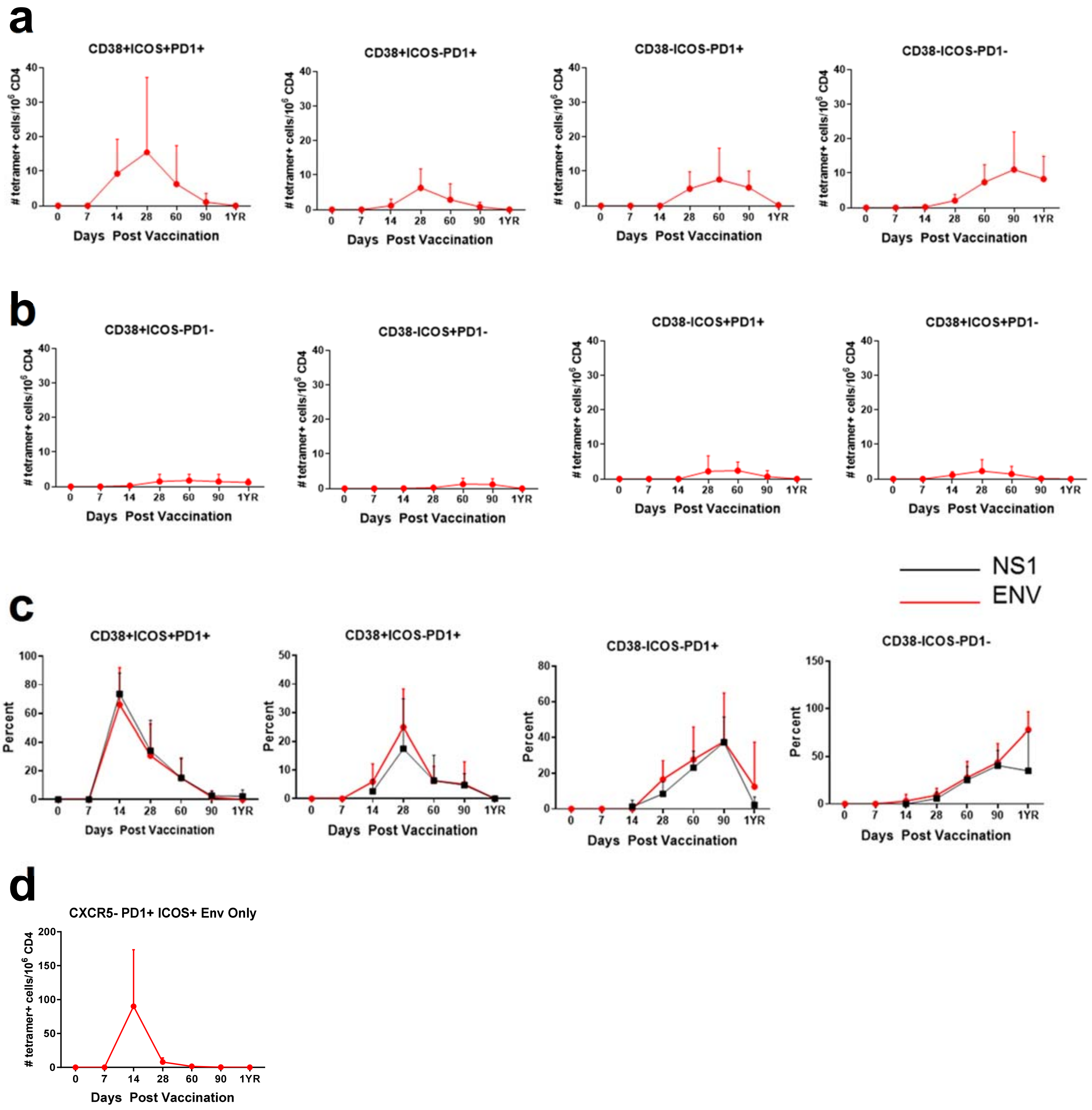
a
CD38+ICOS+PD1+
CD38+ICOS-PD1+
CD38-ICOS-PD1+
CD38-ICOS-PD1-
tetramer+ cells/10^6 CD4
Days Post Vaccination
b
CD38+ICOS-PD1-
CD38-ICOS+PD1-
CD38-ICOS+PD1+
CD38+ICOS+PD1-
NS1
ENV
c
CD38+ICOS+PD1+
CD38+ICOS-PD1+
CD38-ICOS-PD1+
CD38-ICOS-PD1-
Percent
d
CXCR5- PD1+ ICOS+ Env Only
0 7 14 28 60 90 1YR

Table S1: T cell epitopes and tetramer reagents

HLA	Epitope	Amino Acid sequences	Tag
DRB1*0301	YFV ENV 43-59	ISLETVAIDRPAEVRKV	163, 164
DRB1*0301	YFV NS1 85-101	DISVVVQDPKNVYQRGT	168, 169
DRB1*0301	YFV NS3 355-371	WILADKRPTAWFLPSIR	171, 173
DRB1*0301	TT 506-525	NYSLDKIIVDYNLQSKITLP	163, 173
DRB1*0301	EBV EBNA2 301-320	PAQPPPGVINDQQLHHLPSG	168, 175
DRB1*0301	FLU B HA 258-274	GRIVVDYMVQKPGKTGT	169, 171
DRB1*1101	YFV ENV 373-389	DSYIIVGRGDSRLTYQW	PE
DRB1*1501	YFV ENV 457-473	MGAVLIWVGINTRNMTM	PE
DRB1*1101	FLU A HA 161-189	FYKNLIWLVKKGNSYPKLSK	PE
DRB1*1501	FLU A HA 433-452	LDIWTYNAELLVLLENERTL	PE

Table S2: Antibodies used in CyTOF experiments

Antibody	Metal	Vendor	Clone
CD45	89	Fluidigm	HI30
CCR6	141	Biolegend	G034E3
CD19	142	Fluidigm	HIB19
CD45RA	143	Fluidigm	HI100
CD62L	144	Biolegend	DREG-56
CD4	145	Fluidigm	RPA-T4
CD8	146	Fluidigm	RPAT8
CD20	147	Fluidigm	2H7
CCR7	148	Biolegend	G043H7
CD25	149	Fluidigm	2A3
CD3	150	Biolegend	OKT3
CD45RO	151	Biolegend	UCHL1
CD127	152	Biolegend	A01955
CD38	153	Biolegend	HIT2
CD49d	154	Biolegend	9F10
CD27	155	Fluidigm	L128
CXCR3	156	Biolegend	G025H7
CCR4	158	Fluidigm	L291H4
CD161	159	Biolegend	HP3G10
CD14	160	Fluidigm	M5E2
CRTH2	161	Biolegend	BM16
CD39	162	Biolegend	A1
CD122	165	Biolegend	Tu27
CXCR5	166	BD Pharmingen	RF8B2
CD71	167	Biolegend	CYIG4
ICOS	170	Biolegend	C398.4A
OX40	172	Biolegend	Act-35
PD1	174	eBiosciences	eBioJ105

Table S3: Percentage of cells in each of the nineteen different phenotypic cluster

Cluster	EBV	FLU	TT	YFV	Other CD4+	
1	0.55	1.75	4.16	0.67	9.49	Naive
2	1.33	0.58	0.58	0.45	3.46	Treg like
3	0.16	1.34	0.69	0.07	3.96	
4	0.08	0.03	0.07	0	0.06	
5	0.86	0.34	0.22	0.21	0.87	
6	6.8	4.63	1.23	22.22	2.28	Activated
7	4.61	4.67	0.51	50.37	1.02	Activated
8	32.34	57.09	29.74	4.12	5.31	Th1 like
9	2.81	0.48	0.51	1.15	0.16	
10	28.98	8.72	3.29	15.72	2.13	cCXCR5 like
11	1.95	0.45	16.15	0.31	3.19	Th17 like
12	6.25	6.18	33.86	1.13	6.94	Th17 like
13	3.83	0.58	0.72	0.28	5.73	Naive
14	0.94	0.03	0.14	0.22	0.39	
15	1.09	0.76	1.26	0.34	0.98	
16	0	0.07	0	0.03	0.03	
17	5.55	7.62	2.2	1.17	7.07	cCXCR5 like
18	1.62	2.06	2.71	0.98	7.63	Th2 like
19	0.23	2.61	1.95	0.57	39.31	Naive

Table S4. Percentage of YFV-specific cells across the nineteen phenotypic clusters at different time point

Cluster	Day 0	Day 7	Day 14	Day 28	Day 60	Day 90	1 year	
1	20.25	5.19	0.15	0.88	0.79	1.45	6.33	Naive
2	3.8	5.19	0.22	0.56	0.74	1.02	0.32	Treg like
3	1.27	0	0.04	0	0.19	0	0.95	
4	0	0	0	0	0	0	0	
5	0	0	0.06	0.46	0.32	0.68	0.95	
6	5.06	6.49	18.76	36.88	25.54	20.85	6.33	Activated
7	6.33	51.95	74.83	16.7	3.75	2.55	0.32	Activated
8	12.66	3.9	0.26	5.72	10.46	13.19	46.52	Th1 like
9	2.53	1.3	0.89	1.75	1.8	1.02	0.32	
10	6.33	3.9	3.57	30.81	46.97	40.77	8.23	cCXCR5 like
11	2.53	1.3	0.12	0.46	0.32	1.36	0.63	Th17 like
12	2.53	4.55	0.34	1.58	2.5	3.66	3.8	Th17 like
13	10.13	0.65	0.04	0.39	0.88	0.09	1.58	Naive
14	3.8	4.55	0.04	0.14	0.51	0.85	0.32	
15	5.06	4.55	0.21	0.53	0.23	0.51	0	
16	2.53	0.65	0	0	0.05	0.09	0	
17	3.8	1.95	0.07	0.53	2.13	6.89	16.46	cCXCR5 like
18	2.53	1.3	0.25	1.72	2.13	3.49	2.22	Th2 like
19	8.86	2.6	0.14	0.91	0.69	1.53	4.75	Naive
